# Single cell on-chip whole genome amplification via micropillar arrays for reduced amplification bias

**DOI:** 10.1371/journal.pone.0191520

**Published:** 2018-02-12

**Authors:** Harvey C. Tian, Jaime J. Benitez, Harold G. Craighead

**Affiliations:** School of Applied and Engineering Physics, Cornell University, Ithaca, NY, United States of America; Texas A&M University College Station, UNITED STATES

## Abstract

Single cell whole genome amplification is susceptible to amplification biases that impact the accuracy of single cell sequencing data. To address this, we have developed a microfluidic device for the isolation and purification of genomic DNA from individual cells. The device uses a micropillar array to physically capture single cells and its chromosomal DNA upon extraction. The extracted DNA is immobilized within the micropillar array in a way that allows isothermal amplification. In this system, whole genome amplification of the single cell is carried out under a continual fluid flow within the microfluidic channel. We have demonstrated the process for amplification of individual human cancer cell genomes from the HeLa cell line. By sampling select gene loci along the human genome and performing whole exome sequencing, we demonstrate improved genome coverage and reduced amplification bias compared to amplification of single cells deposited in wells by fluorescence activated cell sorting.

## Introduction

Single cell analysis has become increasingly important for understanding and diagnosing disease.[1–6] For instance, cellular level aberrations have been shown to play critical roles in tumor heterogeneity, cancer metastasis, drug resistance, and cell fate.[7–12] Investigating these aberrations and differentiating between cell types within a population may give rise to improved treatments, however, single cell handling and analysis remains difficult. Because there are only picogram quantities of DNA within a single cell, existing due to sensitivity limits, existing workflows cannot sequence single cell genomes directly without amplification.[13–15] Thus, to obtain a sufficient quantity of material for sequencing, single cell whole genome amplification (WGA) is necessary. Among the most widely used single cell WGA amplification techniques is multiple displacement amplification (MDA), which relies on a combination of random sequence primers and the strand-displacement properties of the Phi29 polymerase to isothermally amplify DNA.[14,15] However, amplification bias stemming from chimera formation and non-linear enrichment remain an issue in single cell WGA through MDA.[16–18] This bias can be averaged out when analyzing monodisperse multi-cell population samples because of the multiple copies of each gene from the many cells. However, biases occurring on the single cell level lead to underrepresentation of genome regions that were not amplified early-on in the MDA reaction.[19]

To this end, several techniques have been found to minimize amplification bias during MDA by reducing reaction volumes.[20] Although the mechanism by which reducing amplification volume reduces bias remains to be fully explained, it has been demonstrated across several platforms. These platforms can be broadly categorized into limiting dilution technologies,[21] droplet microfluidic technologies,[22–24] and chambered microfluidic technologies.[19,25] Limiting dilution technologies provide a high degree of parallelism, but the microwells can suffer from cross-contamination of liquids and reagents.[21] More reliable compartmentalization of single cell genomic material can be achieved via emulsion enclosure and microfluidic chambers, however complex channel geometries and valving systems are required to achieve an integrated platform capable of both single cell isolation and genomic analysis. Hence, exploring alternative methodologies of integrating cell capture and genomic analysis is a critical component of the overall effort to improve single cell sequencing.

Recently, our group has developed a valveless microfluidic device for on-chip cell capture and DNA extraction.[26] The approach uses micropillar arrays to physically entrap genomic DNA (gDNA) from cells upon lysis. As this process is purely mechanical, it does not require any chemical modification of surfaces or cell sample preparation.

We have designed a new microfluidic structure for single cell capture and developed a process for genomic amplification via micropillar arrays (GAMA). GAMA relies on the high capture efficiency and DNA immobilization properties of the micropillar array to hold the template gDNA in a fixed position within the microchannel during processing. The genomic DNA is purified in the system because all lysed cell components, including mitochondrial DNA, are washed away while the gDNA is retained in the pillars. This approach also differs fundamentally from existing technologies in that our microfluidic chamber containing the tethered template DNA is subjected to a constant hydrodynamic flow throughout the WGA process. This constant flow also allows reagents within the channel to be continually replenished while amplified product washes downstream into the output reservoirs where it may be collected for off-chip analysis. The purification of the gDNA achieved during the preceding gDNA extraction step also reduces artifacts in the resulting WGA product pool. To characterize our approach, we compare genome coverage through gene loci sampling and whole exome sequencing (WES) between GAMA and FACS based methods.

## Methods

### Cell culture

HeLa-GFP cells (Paul Soloway Lab; Cornell University) were cultured in Dulbecco’s Modified Eagle medium (DMEM) (Invitrogen) within a T75 flask at 37C and 5% CO2. Cell culture medium was supplemented with 10% fetal bovine serum (FBS) (Atlanta Biologicals; Atlanta, GA), 1% (wt) non-essential amino acids (NEAA) (Gibco, Life Tecnologies), 1% (wt) L-glutamine (Gibco, Life Tecnologies), 2% (wt) HEPES (Quality Biological; Gaithersburg, MD), and 0.001% 2-mercaptoethanol (βME) (Sigma-Aldrich; St. Louis, MO). Cells were passaged at 60% (vl) confluency roughly twice per week.

### Device fabrication

Silicon molds for polydimethylsiloxane (PDMS) microfluidic devices were fabricated using standard photolithography techniques. Briefly, wafers (Ultrasil; Hayward, CA) were spin coated with Microposit S1813 photoresist (Shipley; Marlborough, MA). Device pattern was transferred onto photoresist layer using UV contact lithography (ABM contact aligner, ABM-USA; San Jose, CA). Exposed substrates were developed in 726MIF developer (Microchemicals). Microfluidic pattern was transferred onto top silicon layer by Bosch process in a Unaxis SLR 770 deep reactive ion etching system (Unaxis USA Inc.; St. Petersburg, FL). Etch depth was determined to be 20–25 μm using a P10 profilometer (KLA Tencor; Milipitas, CA) and a Zygo otical profilometer (Zygo Corporation; Middlefield, CT). A monolayer of (1H,1H,2H,2H-Perfluorooctyl) Trichlorosilane was deposited on the etched wafers in a MVD100 molecular wafer deposition system (Applied Microstructures; San Jose, CA) to prevent adhesion of PDMS to the mold. Sylgard 184 (Dow Corning; Midland, MI) PDMS base resin was mixed with the curing agent at a 10:1 ratio, degassed under vacuum at room temperature, poured onto the master, and cured for 45 minutes at 150°C. The elastomer casting was then peeled off the mold and access holes to the input and outputs of the microchannels were created with a 1.5 mm biopsy punch (Sklar Instruments; West Chester, PA). To complete channel fabrication, the patterned PDMS was treated with air plasma for 1 minute and bonded to a 500 μm thick fused silica wafer (Mark Optics; Santa Ana, CA). Experiments were run with the microfluidic device mounted on the stage of an Olympus IX-70 inverted microscope (Olympus; Center Valley, PA) to image and observe the microfluidic channels in real time.

### Cell capture and lysis

HeLa-GFP cells were trypsinized from T75 flasks with 0.25% Trypsin. Trypsinized cells neutralized with 1:1 dilution of media, spun down in a centrifuge, and then resuspended in fresh PBS at a concentration of 1:50. The cell suspension was flowed into the microfluidic device via pressure driven flow at 0.5 psi with bone-dry nitrogen gas (Airgas; Radnor Township, PA). The infusion apparatus was then disconnected from the microfluidic device’s input port, washed with alternating cycles of ultrapure water (Invitrogen; Carlsbad, CA) and 100% ethanol to remove the remaining cells within the reservoir, and then reconnected to the microfluidic device input port. Sterile PBS buffer was then flowed into the microfluidic device for 5 minutes to allow uncaptured cells to either be arrested within the cell capture region or to flow through the device into the output reservoirs. The output reservoirs of the device were then emptied and rinsed with ultrapure water. The entire device is visually inspected for any potential cells lodged outside of the micropillar array. Channels that have trapped multiple cells are discarded in post-amplification analysis. Similarly, in cases where a cell has become lodged upstream to the channel divider, the entire device is discarded, and the experiment is ended to prevent inaccurate analysis resulting from contamination. Lysis buffer comprised of 6M guanidinium thiocyanate (Sigma-Aldrich; St. Louis, MO) in water was flowed into the microfluidic device for 5 minutes also by pressure driven flow at 0.5 psi. After visually confirming cell lysis in all ten channels, the lysis buffer was removed from the input reservoir and the entire microfluidic device is flushed with 100% ethanol for 5 minutes. The ethanol is replaced by washing with ultrapure water for 5 minutes and then finally replaced by PBS buffer. The output reservoirs that now contain a mixture of cell lysates, lysis buffer, ethanol, water, and PBS was then emptied and cleaned via rinsing first with 100% ethanol and then ultrapure water. The genomic DNA tethered within the microfluidic device is now ready for whole genome amplification.

### On-chip whole genome amplification

Whole genome amplification (WGA) of the single cell genomic DNA tethered within the micropillar array region of the microfluidic device was carried out using reagents from the REPLI-g UltraFast Mini Kit (Qiagen; Hilden, Germany). Prior to starting the reaction, 280μl of buffer D1 was made by adding 35μl of buffer DLB to 245μl of ultrapure H_2_O. 400μl of buffer N1 was then prepared by adding 40μl of stop solution to 360μl of ultrapure H_2_O. Finally, 288μl of master mix was made by adding 18μl of polymerase to 270μl Repli-G UltraFast reaction buffer. To denature the double stranded gDNA tethered on the micropillar array, buffer D1 was flowed through the device continuously at room temperature for 8 minutes. Buffer D1 was then removed and the device was flushed with buffer N1 for 15 minutes. Afterwards, both the infusion apparatus and the ten output reservoirs were emptied and washed with 100% ethanol and then ultrapure water. The infusion apparatus was then loaded with the master mix solution and pressure was set to 0.5 psi. Pressure was then held constant throughout the entire duration of the 3.5 hour reaction amplification reaction while the device was placed atop a hot-plate set to 33C. After the reaction was completed, 5μl of ultrapure water was added to the amplified DNA product at each output reservoir. The amplified genomic DNA in the reservoir was then pipette collected from each output reservoir off-chip into a polymerase chain reaction (PCR) tube. All samples were heat inactivated at 65C for 10 minutes and placed in a -20C freezer until further use. Sample yield was measured using Qubit 2.0 Fluorometer (ThermoFisher; Waltham, MA) with dsDNA HS dye kit at a 1:200 sample dilution.

### FACS single cell WGA

A FACS machine (Becton Dickinson Biosciences; San Jose, CA) was used to sort single HeLa-GFP cells into a PCR-compatible microwell plate (Bio-Rad; Hercules, CA) with each well containing 5μl of sterile PBS buffer. The microwell plate was then spun down in centrifuge at 1000G for 5 minutes to ensure that sorted single cells were sitting at the bottom of their respective wells. Buffer D2 (buffer D1 supplemented with 10% Dithiothreitol for cell permeabilization) and master mix were then prepared according to the Repli-g UltraFast kit’s protocol. To lyse the single cells in each microwell, 5μl of buffer D2 was added to each well and incubated on ice for 10 minutes. 5μl of stop solution was then added to each well and incubated on ice for 5 minutes. Finally 53.3μl of master mix was added to each well and the microwell plate was placed in a thermocycler (Eppendorf; Germany) set to hold at 30C for 3.5 hours.

### Gene loci PCR

Primers were designed to target 150bp-200bp regions within six gene loci (ERBB2 17q12, PRMT2 21q22, P53 17p13, CCND1 11q13, TRAM1 8q13, and MyC 8q24) and ordered through Integrated DNA Technologies (IDT; Coralville, IA). Lyophilized primers were dissolved in water to a concentration of 10μM. Then, following the protocols from the Taq DNA Polymerase Kit (Life Technologies; Carlsbad, CA), 50μl reaction were prepared for each of the 6 gene loci for every collected single cell WGA sample. 30 cycles of PCR were carried out and the PCR product was run on a 2.3% agarose gel via electrophoresis. Using a 2-log ladder (New England Biosciences; Ipswich, MA), the appropriate size region of 100bp-200bp was evaluated for the presence or absence of the gene.

### Whole exome sequencing

Whole exome sequencing (WES) was performed via the Weill Cornell Medical College sequencing facility. Samples libraries were prepared using Agilent SureSelect Target Enrichment Kit (Agilent; Santa Clara, CA). Exome pulldown was performed using SeqCap EZ Exome v3 Human Exome Kit (Roche Sequencing; Pleasanton, CA). Samples were sequenced in a single lane on a HiSeq 4000 (Illumina; San Diego, CA) with paired end clustering 100x2 cycles. Resulting reads were aligned to human reference genome HG19.

## Results and discussion

### Channel design and experimental setup

**Fig 1:**
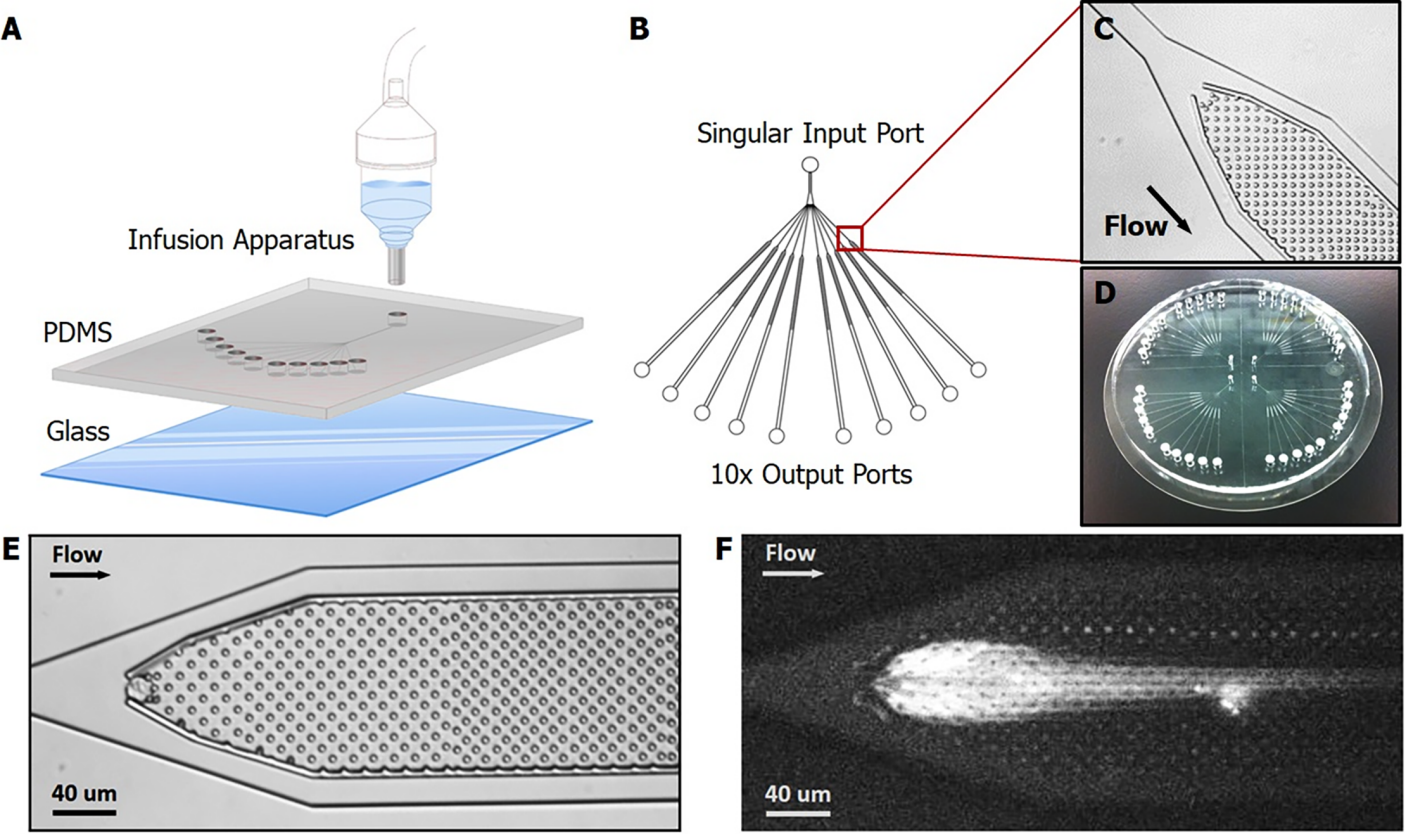
Experimental setup, device design, and in-channel visualization. (A) Exploded view of our PDMS microfluidic device. An infusion apparatus is connected to the single input port to provide pressure driven flow of fluids and reagents. (B) Device schematic showing valveless 10-channel device design in which product from each channel can be collected separately. (C) A micrograph of the single cell capture region and micropillar array. (D) Picture taken of 4 separate 10-channel devices made from a single PDMS slab bonded to a 4-inch diameter glass silica wafer. (E) Micrograph showing single cell capture, and (F) subsequent lysed cell imaged under fluorescence with YOYO-1 intercalating dye staining of genomic DNA immobilized within the pillar array region.

Fig 1(A) shows the overall experimental setup for GAMA. To create the chip device, a slab of mold-casted PDMS (polydimethylsiloxane) imprinted with the channel geometry is bonded to a glass slide to create the microfluidic device. Reagents are loaded into the device via pressure driven flow from an infusion apparatus housing a large fluid reservoir. Fluid that is loaded into the infusion reservoir can be easily exchanged and replaced via pipetting. The infusion apparatus is a two-part mechanism consisting of a reservoir portion that can be connected to the microfluidic device via an input port and a cap that is connected to a nitrogen source used to drive channel flow. Fig 1(B) shows a top down view of the device design. The GAMA device has a single input port and 10 separate output ports allowing multiple single cell samples to be run in parallel. These ten channels each contain identical designs consisting of a single cell capture region and micropillar array Fig 1(C). HeLa cells expressing green fluorescent protein (GFP) were selected as a model cell type for our experiments due to the ease of handling and fluorescent properties. To show the device in scale, Fig 1(D) shows four such devices can be casted from a 4-inch silicon wafer mold as a single slab and bonded to a glass-silica wafer.

### Single cell GAMA

As depicted by the graphic in Fig 1(E), the single cell capture region consists of a series of 1.5μm posts spaced 2μm apart and arranged in an orientation to arrest cells in the apex of the micropillar array. The size and spacing of these posts were selected to be small enough so that the genomic chromosomal DNA from the trapped cell would become entangled within the array due to their length while smaller molecules such as RNA, proteins, and lipids would flow through the array unhindered. The height of the microchannel is adjusted to the size of the cells to prevent multiple cells from stacking on top of one another in the Z-plane. Furthermore, barriers enclosing the micropillar array also aid in preventing cells from lodging in the array downstream of the desired capture region. Occasionally, multiple cells are captured within the micropillar array or become adhered to the glass surface within the microchannel. To reduce cell adhesion on the channel surfaces, we increased flow rate during cell loading to be above 2μl min^-1^. This flow rate was then reduced once the cells were loaded and prior to cell lysis to 0.5μl min^-1^. Channels containing multiple cells are discarded in the analysis and the entire device is visually inspected prior to cell lysis to ensure that there are no cells lodged outside of the desired cell capture region.

Upon introduction of lysis buffer, the micropillar array will physically immobilize the gDNA. This immobilization process occurs as a result of the chromosomal DNA being physically entangled on the pillars due to their centimeter scale lengths, while smaller cellular components such as lipids, proteins, RNA, and mDNA are washed away downstream. The immobilized gDNA can be imaged via fluorescent staining with DNA intercalating dye labels such as with YOYO-1 shown in Fig 1(F).

**Fig 2:**
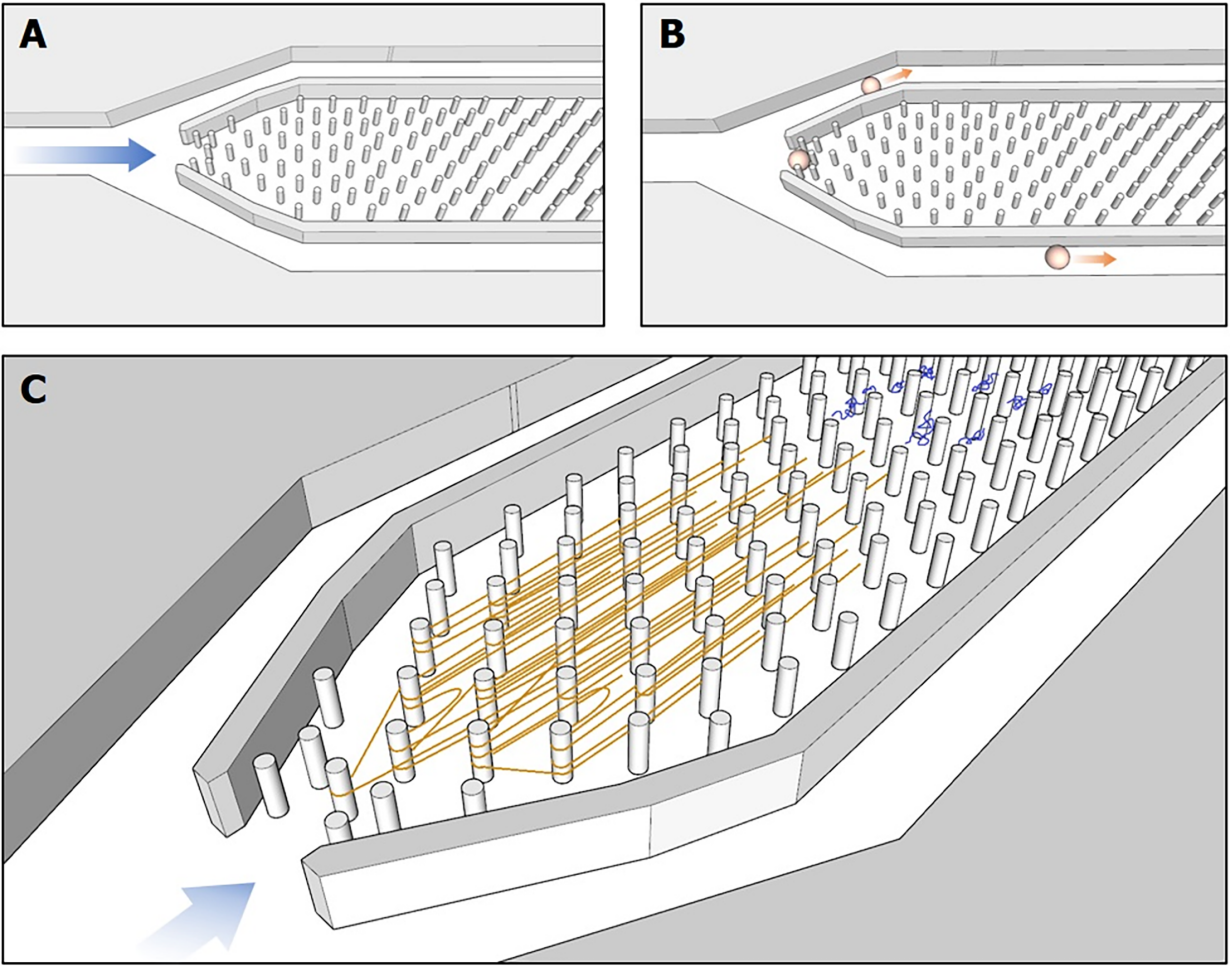
GAMA workflow. Here, a view of the inside of the sealed microfluidic platform is shown. (A) Fluid flow (blue arrow) moves from left to right in this schematic and fluids can be exchanged within the input reservoir to control the local environment within the channels. (B) Artist depiction of a single cell that is trapped within the apex of the micropillar array. Side walls enclosing the micropillar array guide the cell towards the apex of the array while excess cells bypass the pillar array to the output reservoir. (C) Upon cell lysis, genomic DNA (orange) from the trapped cell will become entangled in downstream pillars while debris from the cell lysate is washed away. Reagents for whole genome amplification is flowed into the channel and as amplification occurs, product DNA strands (blue strands) are carried downstream and collected in output reservoirs.

After cell capture and lysis, the extracted gDNA tethered within the micropillar array was amplified via MDA under a continuous flow of 0.5μl min^-1^. An illustration of the GAMA workflow can be seen in Fig 2. Although we had initial concerns that denatured DNA strands would rapidly reanneal prior to amplification, we found GAMA to yield close to a 10,000-fold amplification as measured by final DNA concentration of the amplified product. Another concern was that the highly branched structures characteristic of isothermal amplification with Phi29 would become entangled within the pillar array and occlude the flow, however we did not observe any product buildup or clogging upon inspection with DNA fluorescence imaging post-amplification. We reason that this is because the average sized fragments produced from the MDA, roughly 12kb in length, are too short to tether around the 1.5μm diameter micropillars without slipping off.

### Validation and gene loci detection

Because the MDA kit we used has been shown to produce artifact DNA, simply quantifying the amount DNA collected from the output reservoirs is insufficient to determine the success or failure of on-chip single cell GAMA. Hence, to validate the GAMA technique, 6 different cancer gene loci along the human genome were selected as sampling intervals to ensure successful amplification of that genome region. Using the product collected from GAMA as a template for PCR, the presence or absence of each of the 6 gene loci was evaluated as an initial means of assessing GAMA performance.

**Fig 3:**
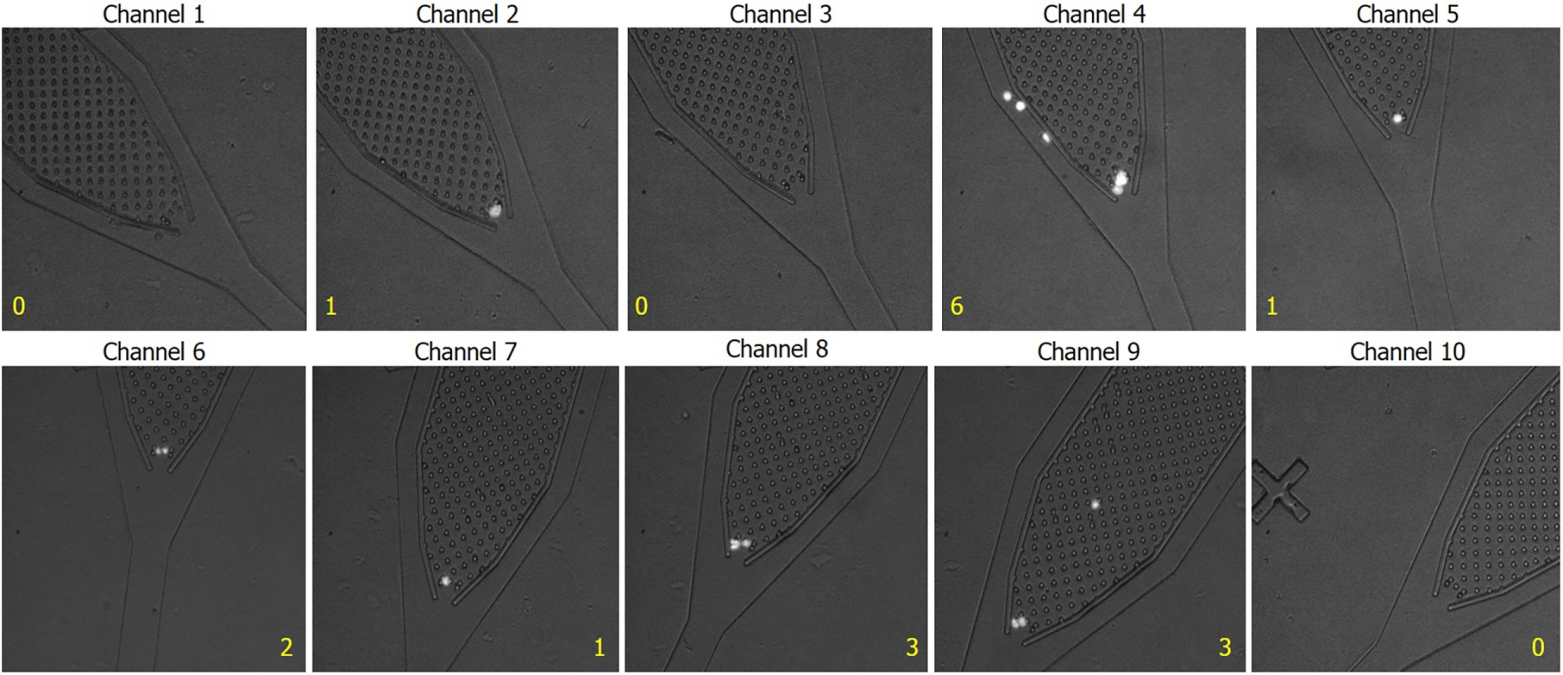
Compiled series of micrographs taken from the cell capture region of a single 10-channel device. Cell counts for each channel are labeled in yellow text. The device is visually inspected for unaccounted cells lodged outside of the pillar array and channels containing multiple cells are discarded. Channels containing single cells (2, 5, &7) are analyzed in comparison to empty channels (1, 3, &10), which serve as negative controls from the same device to further assure the WGA product from each channel does not contain any potential upstream DNA contamination.

**Fig 4:**
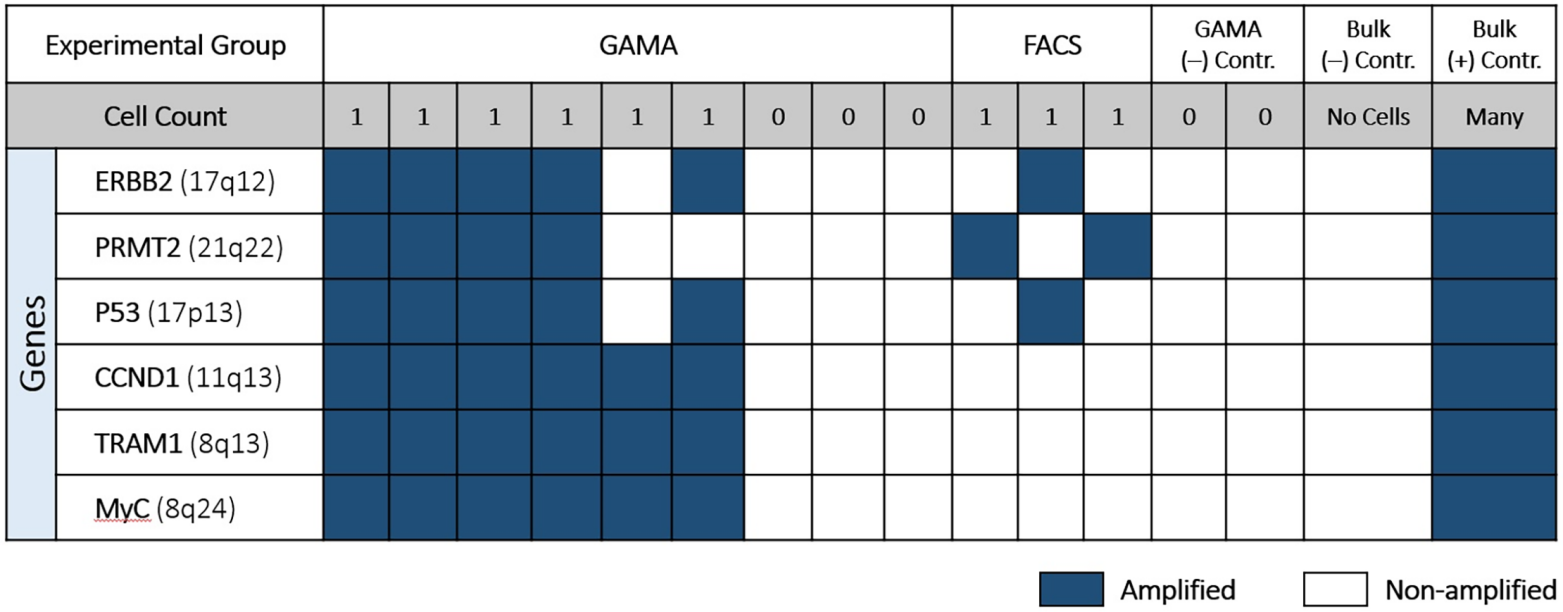
Compiled table of genome coverage analyzed by detection of 6 cancer-relevant gene loci. Using GAMA, single cell WGA covers up to 6/6 targeted gene loci versus 2/6 when using conventional single cell WGA with cell isolated via FACS. In-channel single cell negative controls with no cells expectedly show no gene loci detected, and off-chip bulk-level analysis affirms the specificity of primers used in loci detection.

Fig 3 shows micrographs taken from the cell capture region of the 10 channels in a single device. Channels containing a single HeLa-GFP cell (2, 5, and 7) were compared to channels with 0 cells (1, 3, and 10) in the number of gene loci detected post GAMA. Samples from channels such as channels 4, 6, 8, and 9 are disregarded due to having multiple cells. The capture of multiple cells in certain channels are due to unoptimized channel dimensions and micropillar spacing for the cell type used and occasional occurrences of cell adhesion onto the glass surface due to non-specific binding. In future iterations of the device, non-specific cell adherence can be mitigated through treating the glass surface with blocking agents such as bovine serum albumin (BSA) protein buffers or charge-shielding the channel with chemicals such as polyvinylpyrrolidone (PVP) or polyethylene glycol (PEG). While it was found that many of the gene loci were present in the amplification product collected from single cell channels, as shown by Fig 4, no gene loci were detected in empty channels on the same device. Thus, this indicates that there is no cross-channel contamination or upstream contamination contributing towards our results.

### Comparing GAMA to FACS

In order to assess amplification bias and overall genome coverage we compared gene loci detection between single cells amplified by GAMA to single cells amplified with conventional assays. For the conventional assay route, we used Fluorescence Activated Cell Sorting (FACS) to isolate single cells into PCR-compatible 96-well plates and amplified the gDNA from these cells using identical kits, reaction times, and reagent batches as those used in GAMA experiments. Our findings, shown in Fig 4, is that when using the same reagents and WGA parameters as single cells amplified by GAMA, only one to two gene loci were successfully amplified from the FACS isolated single cells versus 4 to 6 loci successfully amplified by GAMA.

**Fig 5:**
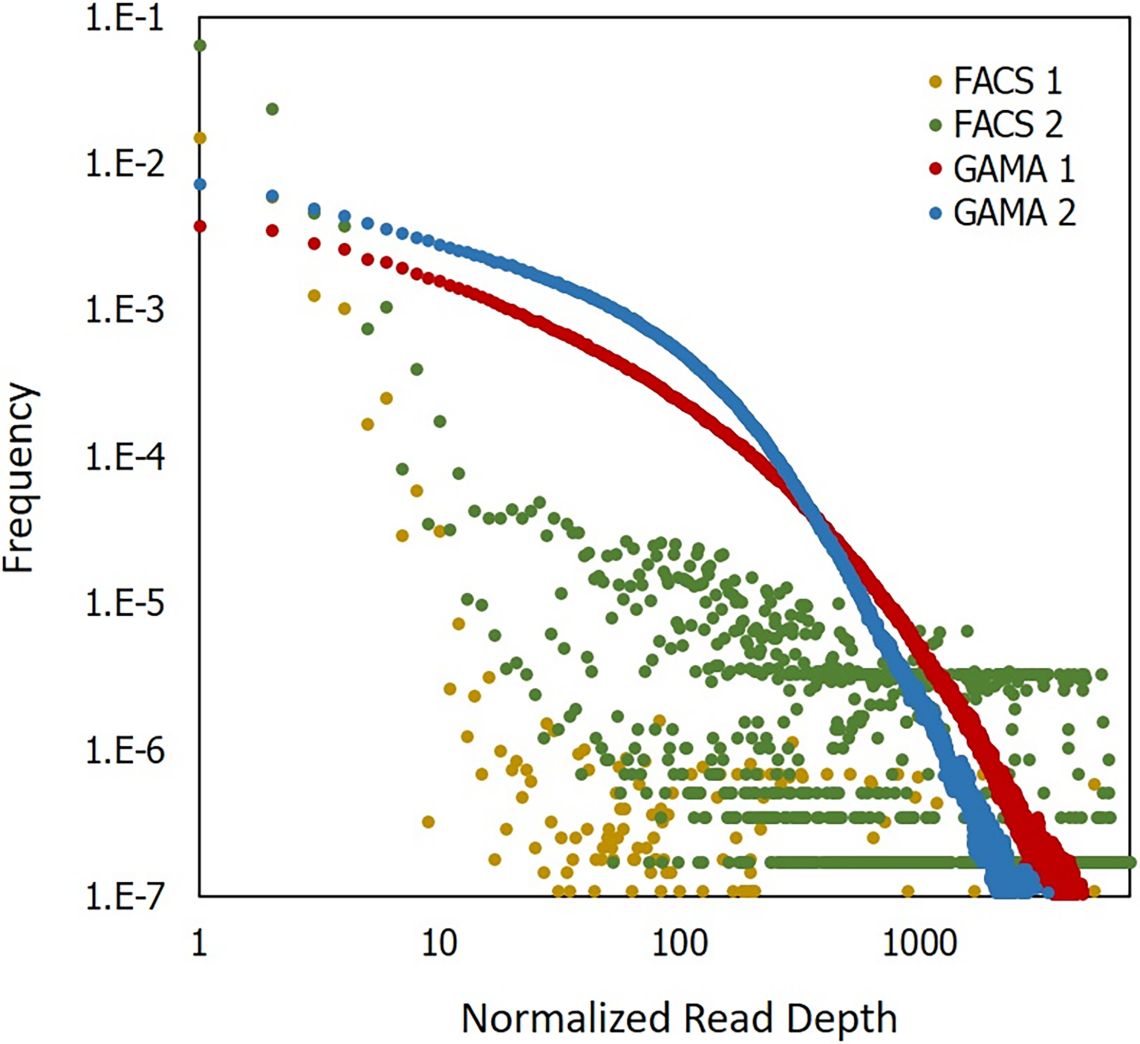
Characterizing the amplification uniformity of GAMA. The two FACS isolated single cell samples whole genome amplified by MDA exhibit more of low depth reads with a sharp drop off in read count before reaching 10x coverage. In contrast, GAMA single cell samples showed no significant drop in frequency of reads as depth increased and showed more uniformity across the genome.

Further expanding upon the gene loci detection results, we submitted single cell whole genome amplified samples from both GAMA and FACS for WES. In sequencing, we found that single cells amplified by GAMA had a higher percentage of mapped reads compared to single cells isolated through FACS and amplified by conventional assays (highest percentage of mapped reads 98.5% for GAMA versus 65% for FACS). Additionally, as shown in Fig 5, we found that although FACS samples had a higher frequency of low depth (≤3) reads compared to GAMA samples, FACS samples experienced a sharp drop off in read count as depth increased. In contrast, GAMA samples exhibited a much more gradual decline in read count as a function of depth. This suggests that the GAMA process amplifies single cell genomes with higher uniformity than conventional WGA assays. However, as we maintained identical reagents and reaction times between FACS samples and GAMA samples, we attribute the differences in amplification uniformity to a few key differences between GAMA and conventional WGA assays.

The first of these advantages is in performing amplification under a constant flow. Doing so allows amplification reagents to replenish the local template environment more rapidly than diffusion dependent systems. Flow higher than this add no reaction advantage, but sufficiently high flows could shear or dislodge the DNA from the pillars. Our selectin of flow rate in this stage of the process included considerations of minimizing the use of costly reagents. Moreover, another advantage conferred by performing amplification under a constant flow is that the amplified product DNA is carried downstream by fluid flow away from the immobilized template once the polymerase detaches from the template, thereby possibly reducing or prevent chimera formation. Secondly, as the template gDNA is linearized throughout the amplification process in GAMA, polymerase molecules have increased overall accessibility to the template whereas the gDNA in conventional assays would be in a coiled conformation thereby biasing towards binding of polymerase on the gDNA away from the center of the coil. Finally, the third advantage of performing single cell WGA with GAMA lies in the micropillar based cell lysis and DNA extraction process. As the GAMA cell lysis process uses size-based selection to physically capture gDNA within the micropillar array while smaller molecules such as lipids, proteins, RNA, and mDNA are filtered out of our device, GAMA essentially provides a purification of the gDNA template prior to WGA. In our comparisons, we found FACS samples to contain over two orders of magnitude more mDNA than GAMA samples (>20,000 mDNA reads for FACS; < 150 mDNA reads for GAMA).

Although whole genome sequencing will be required to further characterize single cell GAMA, the overall coverage breadth of GAMA for all targeted exomes (81.08% at ≥1x depth; 70.35% at ≥10x depth) was comparable to existing single cell WGA methods such as Multiple Annealing and Looping Based Amplification Cycles (MALBAC) and Emulsion Whole Genome Amplification (eWGA).[23] However, as we did not carry out independent WES trials using MALBAC or eWGA, we refrain from venturing conjectures towards the source of differences between these methods and GAMA.

## Conclusion

We have described a simple, valveless, and scalable micropillar-based microfluidic device capable of on-chip single cell processing and WGA. Unlike conventional single cell platforms, the GAMA platform performs WGA under constant flow on purified and immobilized gDNA. Although we have yet to optimize these flow rates and their effects on the final amplification performance, we have taken the first step to validate the viability of the GAMA approach by demonstrating reproducible on-chip amplification without upstream or cross-channel contamination. Through gene loci PCR, we report that GAMA reliably amplifies more regions of the genome from single HeLa cells as compared to conventional assays with single cells isolated through FACS. Finally, we determined by WES that GAMA has improved amplification uniformity across the genome and greatly reduced levels of non-specific amplification, such as amplification of mDNA. Together, these findings suggest GAMA may be used as a platform to overcome current bias-based limitations experienced by conventional single cell assays and improve the accuracy of single cell analysis.

## Supporting information

S1 File Primer sequences used in gene loci detection. (PDF)

S2 File Gene loci detection gel images. (PDF)
