# Supplementary material for: Single cell on-chip whole genome amplification via micropillar arrays for reduced amplification bias
Source: PLoS One. 2018 Feb 12;13(2):e0191520. doi: 10.1371/journal.pone.0191520 (PMC5809021; doi:10.1371/journal.pone.0191520)
Supplement: S1 File — (PDF) [file pone.0191520.s001.pdf]

Single cell on-chip whole genome amplification via micropillar arrays for reduced amplification bias

Supporting information

Loci	Forward Primer Sequence	Reverse Primer Sequence
CCND1	CCATTCCATTTCCAAGCACTTTCAGTCC	GTGGCCCACACTTGCCTCAA
MyC	CTCCACTCTCCCTGGGACTCTTG	CCTGTGAGTATAAATCATCGCAGGCGG
PRMT2	CCAAACTTCCTCTCCCCACAACAGA	GCGACAACCCCTACGAGCCACAA
P53	GTCTGAACGCTTCTATCTTGGCGAGAA	GGAGACAGGTCTGAAGCCTGGA
TRAM1	CCAGGTTTGGGCAAAACGATAAGAGGA	CACTGGCCTCAGACTGAAGACGAA
ERBB2	CTTTGCCCACTATGGTCCAAATCGA	GCAGAGAATGATTTGAGCCCCTGAAAGA

S1 Table. Primer sequences used in gene loci detection.
