# Supplementary material for: Single cell on-chip whole genome amplification via micropillar arrays for reduced amplification bias
Source: PLoS One. 2018 Feb 12;13(2):e0191520. doi: 10.1371/journal.pone.0191520 (PMC5809021; doi:10.1371/journal.pone.0191520)
Supplement: S2 File — (PDF) [file pone.0191520.s002.pdf]

Single cell on-chip whole genome amplification via micropillar arrays for reduced amplification bias

Supporting information

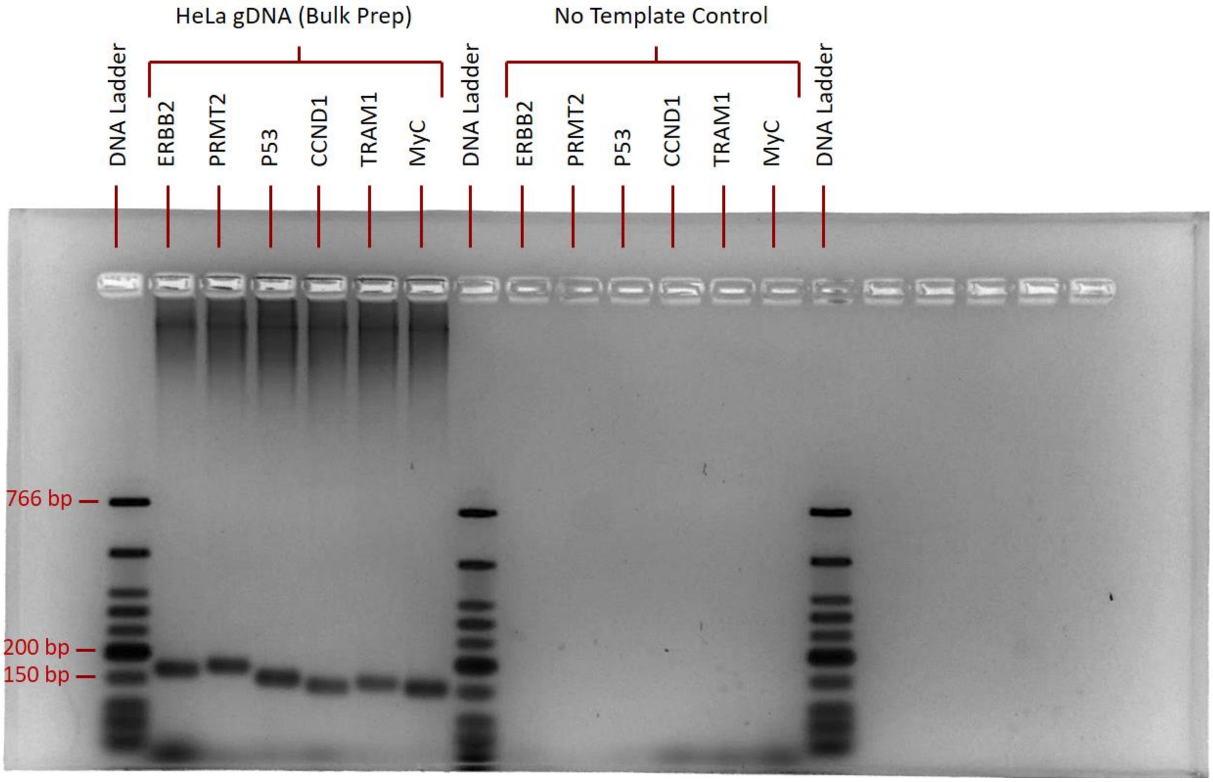

S2 Figure. Off-Chip Gene Loci PCR Control Using Bulk-Extracted HeLa gDNA.

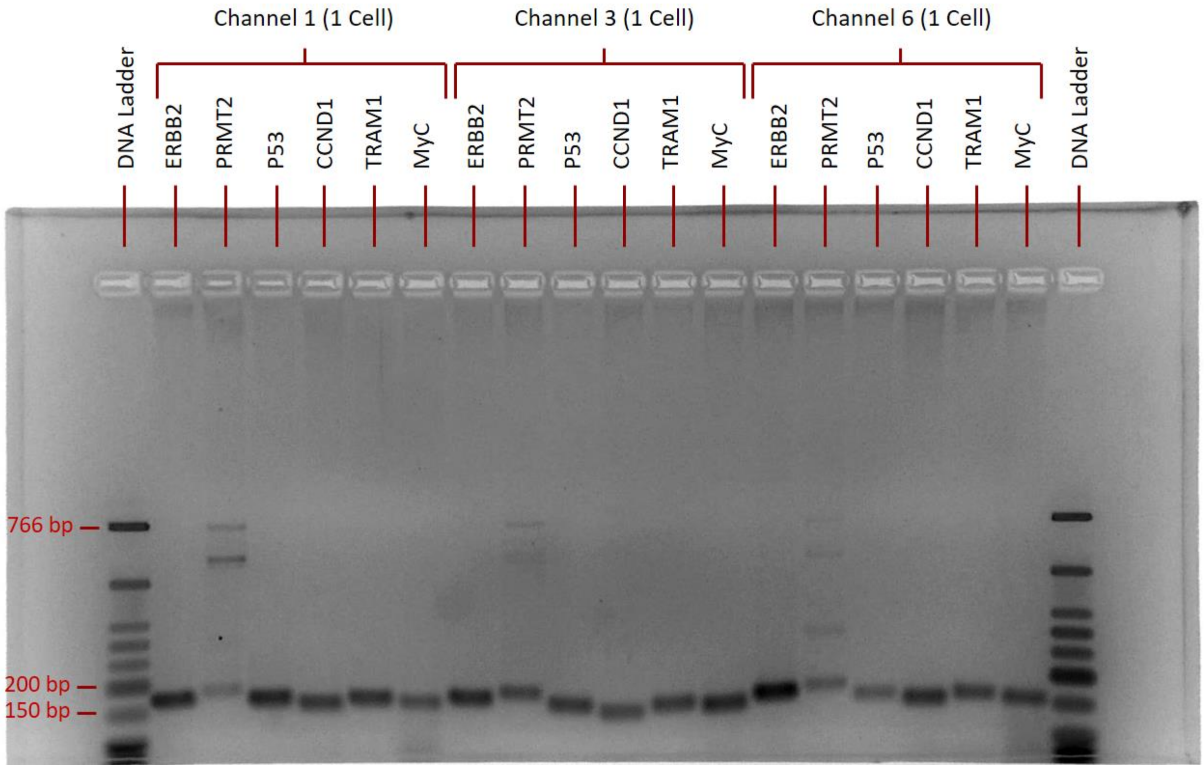

S2 Figure. 6 Gene Loci PCR of 3 Different Single HeLa Cell On-Chip GAMA Collections.

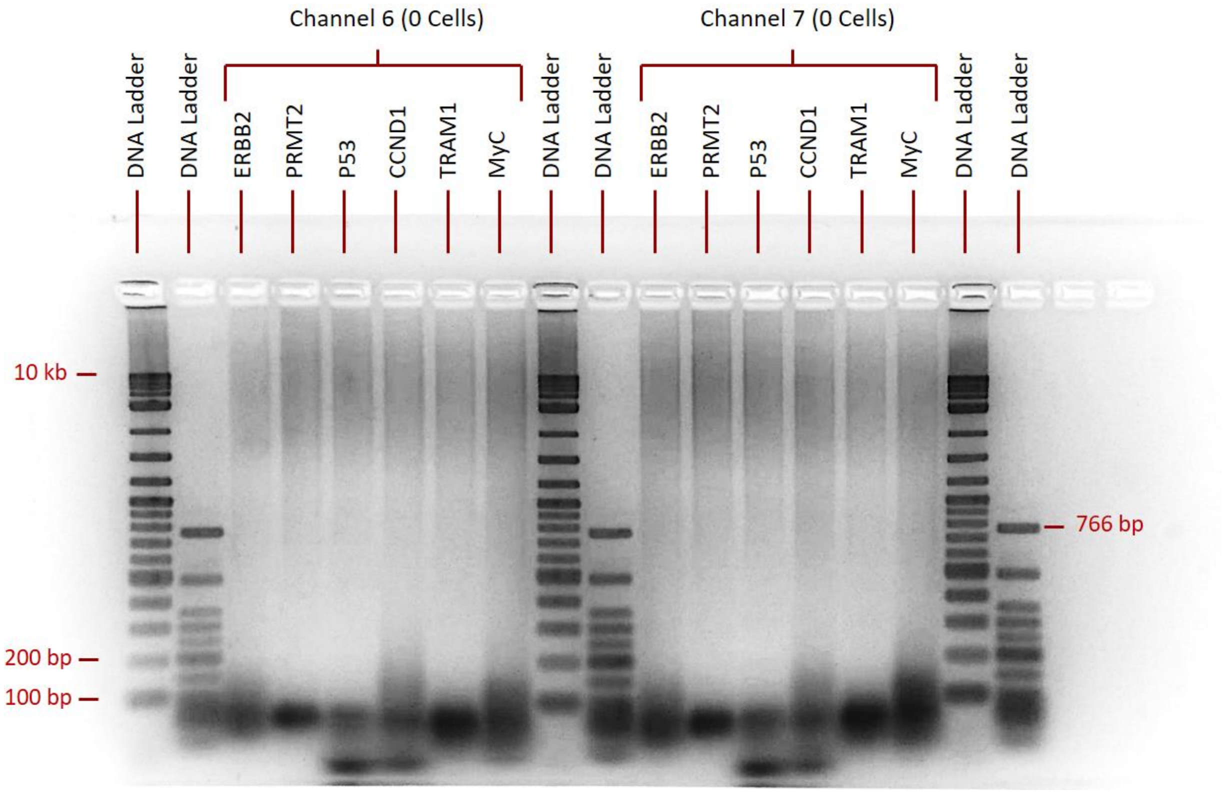

S2 Figure. On-Chip GAMA No-Template Control with Produce No Detectable Gene Loci.

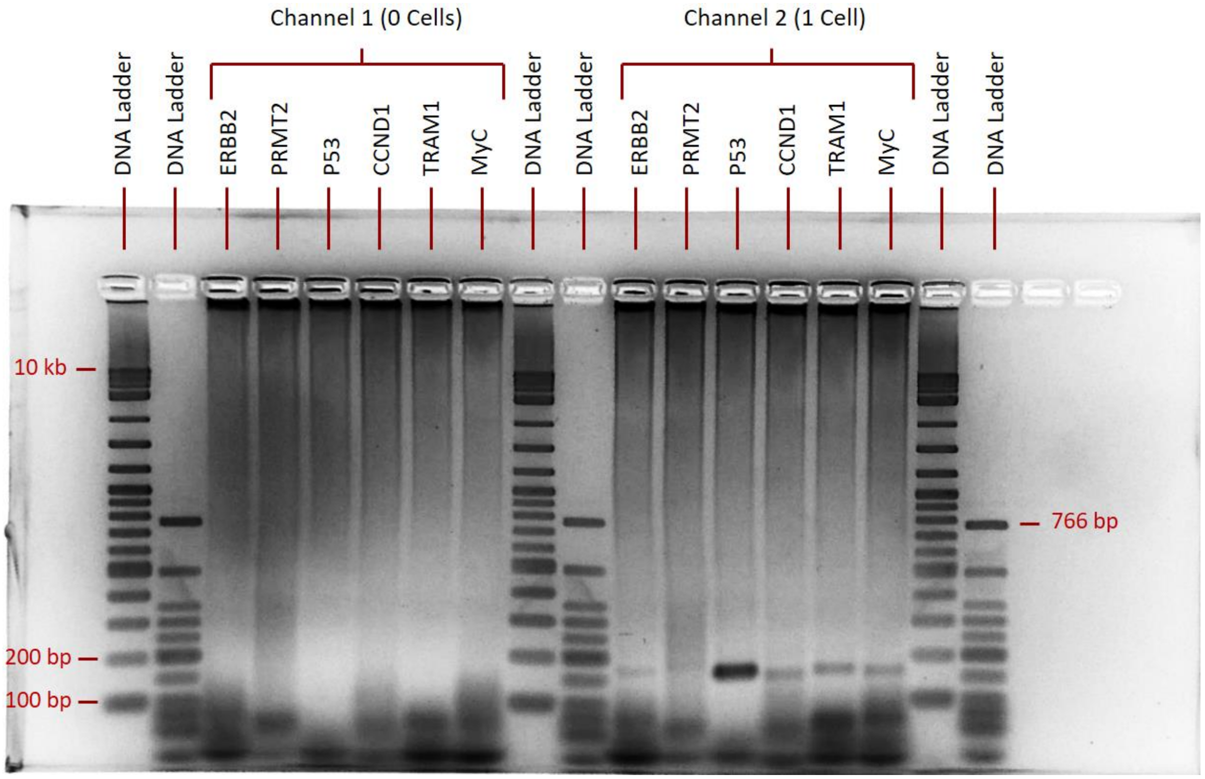

S2 Figure. Gene Loci PCR of On-Chip GAMA Collection of Two Separate Channels Containing 0 Cells and 1 Cell.

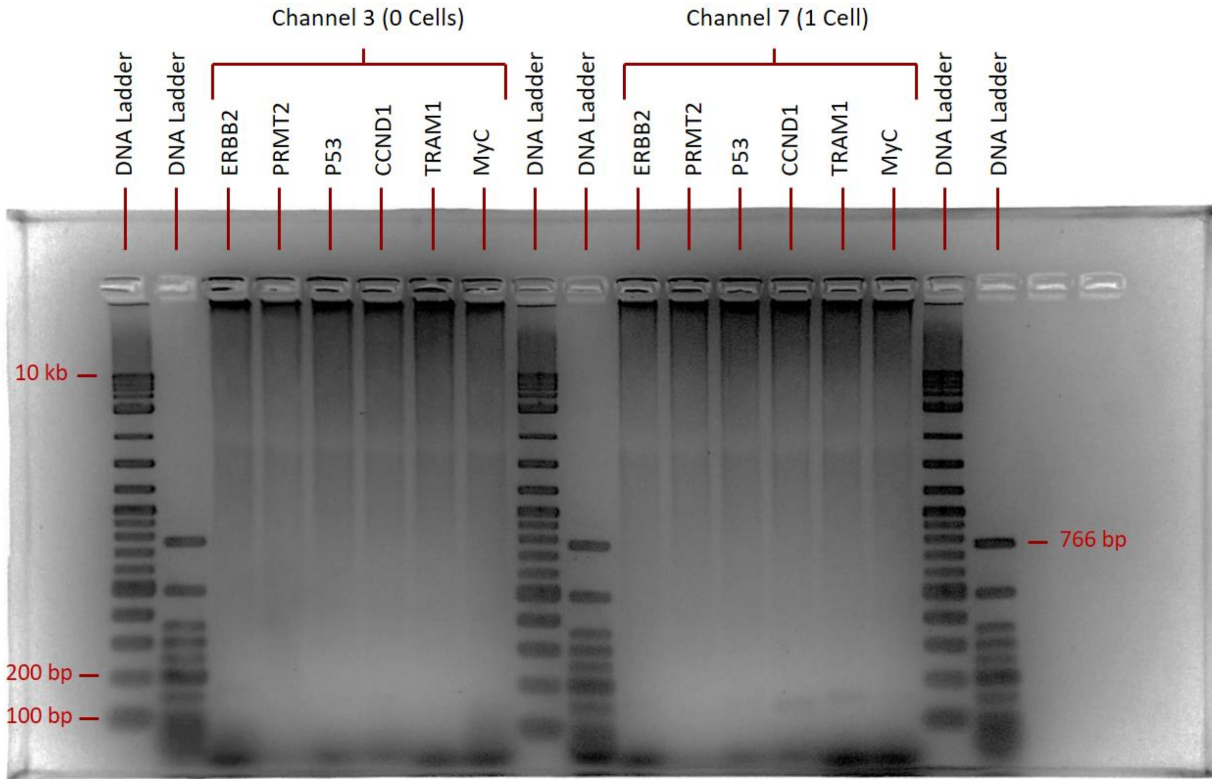

S2 Figure. Gene Loci PCR of On-Chip GAMA Collection of Two Separate Channels Containing 0 Cells and 1 Cell.

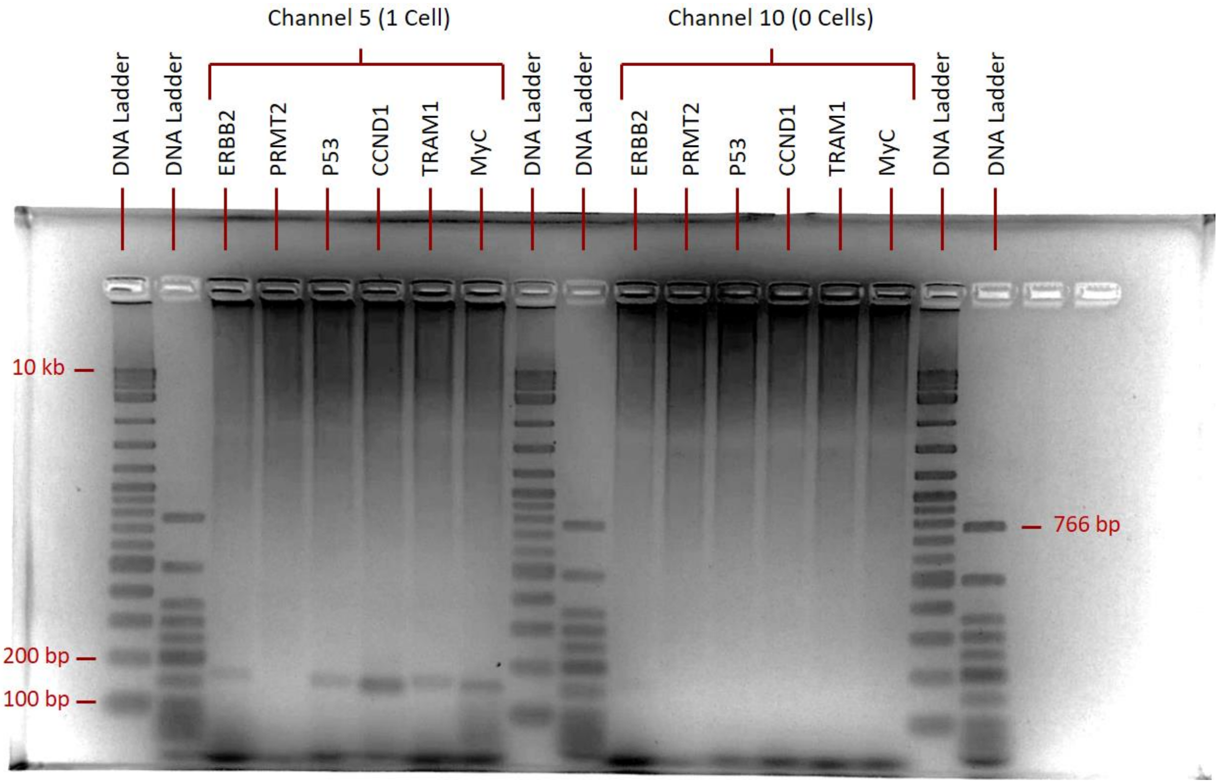

S2 Figure. Gene Loci PCR of On-Chip GAMA Collection of Two Separate Channels Containing 1 Cell and 0 Cells.

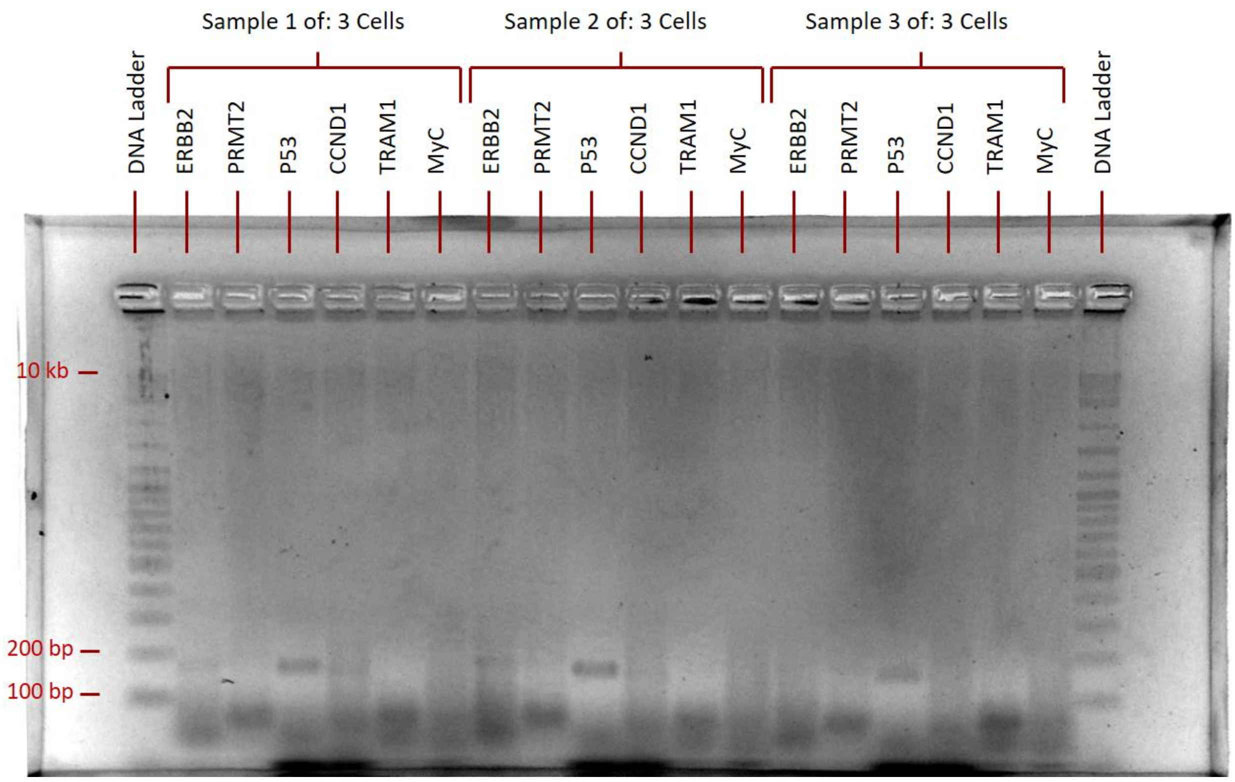

S2 Figure. Gene Loci PCR of 3 Separate Samples of FACS Isolated Single Cells Amplified by MDA Off-Chip.
